# Impact of Sleep Quality, Sleep Disturbances on Quality of Life Among Medical Students Worldwide: A Narrative Review

**DOI:** 10.7759/cureus.108356

**Published:** 2026-05-06

**Authors:** Siddesh Nagabhushan, Mohit Kumar, Somlata Ghosh, Nargiza Kadirova

**Affiliations:** 1 Department of Internal Medicine, Tashkent State Medical University, Uzbekistan, Tashkent, UZB; 2 Department of Family Medicine and Preventive Medicine, Tashkent State Medical University, Uzbekistan, Tashkent, UZB

**Keywords:** academic performance, medical students, quality of life, sleep deprivation, sleep disorders, sleep quality

## Abstract

Sleep is a fundamental biological process essential for physical health, cognitive performance, and psychological well-being. Medical students represent a particularly vulnerable population for sleep disturbances due to demanding academic workloads, irregular schedules, psychological stress, and lifestyle-related factors. This narrative review synthesizes current evidence regarding the prevalence of sleep disorders among medical students, the key risk factors contributing to impaired sleep quality, and the resulting impact on multiple domains of quality of life, including physical health, mental well-being, cognitive function, academic performance, and social functioning. A comprehensive literature search was conducted across PubMed/MEDLINE, Scopus, Web of Science, and eLIBRARY.ru for peer-reviewed studies published between 2010 and 2025. The available literature consistently demonstrates a high prevalence of poor sleep quality among medical students, frequently exceeding 60%, with strong associations with anxiety, depression, burnout, reduced academic performance, and diminished overall quality of life. Early identification through validated screening tools and implementation of institutional interventions, including sleep health education and counseling, may be beneficial strategies to help mitigate potential long-term health and professional consequences.

## Introduction and background

Sleep is a core physiological process essential for maintaining physical health, emotional regulation, and optimal cognitive functioning across the lifespan. Sleep quality is a multidimensional concept that includes sleep continuity, latency, timing, regularity, restorativeness, and daytime functioning [1]. Good-quality sleep is characterized by timely sleep onset, minimal awakenings, restorative sleep, and preserved daytime alertness, whereas poor-quality sleep commonly involves delayed sleep onset, fragmented or nonrestorative sleep, and daytime fatigue. Sleep quality is commonly assessed using validated tools such as the Pittsburgh Sleep Quality Index (PSQI), where scores >5 frequently indicate poor sleep quality [2]. In this review, sleep disturbances refer broadly to problems such as insufficient sleep, frequent awakenings, irregular sleep schedules, or insomnia symptoms, whereas sleep disorders refer to clinically diagnosed conditions such as insomnia disorder, obstructive sleep apnea, or circadian rhythm sleep-wake disorders. Thus, the term poor sleep quality refers primarily to self-reported dissatisfaction with sleep characteristics such as duration, continuity, restorativeness, or daytime functioning, commonly assessed using instruments such as the PSQI. Poor sleep quality among medical students has been increasingly associated with impaired well-being, psychological distress, and lower academic performance [3-5].

Among clinically recognized sleep disorders, insomnia disorder is one of the most common and is characterized by persistent difficulty initiating sleep, maintaining sleep, or early morning awakening accompanied by daytime impairment [1]. However, beyond diagnosed sleep disorders, many medical students experience nonclinical sleep-related problems such as chronic sleep deprivation, defined as habitual insufficient sleep occurring repeatedly over weeks or longer, with sleep duration consistently below individual physiological needs; circadian rhythm misalignment, characterized by a mismatch between endogenous biological timing and externally required schedules, often presenting as difficulty sleeping at desired times or excessive daytime sleepiness; and irregular sleep-wake patterns, marked by inconsistent bedtimes and wake times, substantial day-to-day variability, or frequent weekday-weekend schedule shifts [1,6]. These patterns are particularly relevant in medical training, where academic demands, examinations, and clinical duties may contribute to reduced sleep duration and poorer sleep quality.

University students represent a population at an increased risk of sleep problems due to major transitions in daily routines, living arrangements, and social environments, combined with heightened academic demands. Medical students, in particular, experience additional stressors, including prolonged study hours, frequent examinations, early clinical exposure, night duties, and high expectations for academic and professional performance. The cumulative effect of these stressors often results in irregular sleep schedules, maladaptive sleep behaviors, and chronic sleep restriction. Consequently, sleep disturbances are highly prevalent among medical students and constitute an important yet frequently under-recognized public health concern within medical education [3,4].

Medical students frequently experience prolonged study hours, examination-related stress, early morning academic schedules, and, during clinical training, night duties or extended hospital responsibilities, all of which may adversely affect sleep patterns [3,4,7]. Given the high prevalence of poor sleep quality reported among this population, this narrative review aimed to synthesize current evidence regarding sleep quality and related sleep disturbances among medical students, identify major contributing factors, and evaluate their impact on quality of life across physical, psychological, academic, and social domains.

## Review

Literature search strategy

This study was designed as a narrative review to synthesize current evidence regarding sleep quality, sleep disturbances, and quality of life among medical students. A structured literature search was performed using PubMed/MEDLINE, Scopus, Web of Science, and eLIBRARY.ru for studies published between January 2010 and January 2025. Search terms included combinations of “sleep quality,” “sleep disturbances,” “medical students,” “insomnia,” “academic performance,” “mental health,” and “quality of life.” Articles published in English were primarily considered. Eligible studies included cross-sectional studies, cohort studies, case-control studies, systematic reviews, meta-analyses, and other observational studies involving medical students. Editorials, letters, duplicate publications, studies unrelated to medical students, and articles lacking relevant sleep or quality-of-life outcomes were excluded. Titles and abstracts were screened for relevance, followed by full-text review of selected articles. Additional sources were identified through manual screening of reference lists. Given the narrative design of this review, no formal risk-of-bias assessment or meta-analysis was performed.

Prevalence of poor sleep quality and sleep disturbances among medical students

Sleep-related problems are highly prevalent among medical students across diverse geographic regions [2,3,5]. Most studies in this population specifically assess poor sleep quality, commonly using PSQI, whereas the broader term sleep disturbances may also include insufficient sleep duration, insomnia symptoms, and irregular sleep schedules. A large meta-analysis reported a pooled prevalence of poor sleep quality at 52.7% among medical students based on PSQI-defined outcomes [3]. Individual studies have reported even higher rates, including 76% among Saudi medical students, where poor sleep quality was also associated with perceived stress and academic factors [4]. Other studies have linked poor sleep quality with burnout and impaired functioning [8]. Although prevalence estimates vary by region, methodology, and PSQI cutoff values, the available evidence consistently indicates that poor sleep quality is common among medical students worldwide. Differences in reported prevalence may partly reflect variation in PSQI cutoff values used across studies, most commonly >5 or ≥5, with some studies using higher thresholds [3,5]. The prevalence estimates from representative PSQI-based studies are summarized in Table 1.

**Table 1:** Prevalence of Poor Sleep Quality Among Medical Students (PSQI-Based Studies) Note: The pooled prevalence of 52.7% was derived from the meta-analysis by Rao et al. [3] and should not be interpreted as a direct average of the studies listed in this table. PSQI: Pittsburgh Sleep Quality Index, N/A: Not available / not reported.

Author (Year)	Country	Study Design	Sample Size	Assessment Tool	Poor Sleep (%)	Cutoff
Rao et al. (2020) [3]	India	Cross-sectional	400	PSQI	60-65%	>5
Almojali et al. (2017) [4]	Saudi Arabia	Cross-sectional	282	PSQI	76%	>5
Binjabr et al. (2023) [5]	Global	Meta-analysis	-	PSQI	52-60%	Mixed
Khaksarian et al. (2020) [9]	Multiple countries	Meta-analysis	-	PSQI	55-65%	Mixed

Factors affecting sleep quality among medical students

Academic Stress and Educational Pressure

Academic pressure represents one of the most consistently identified risk factors for sleep disturbance among medical students [3,4]. Rigorous curricula, prolonged study hours, frequent examinations, and high expectations for academic performance contribute to heightened cognitive and emotional arousal, which may interfere with sleep initiation and maintenance [4]. Evidence suggests a bidirectional relationship between academic stress and sleep quality, whereby poor sleep further impairs concentration, learning capacity, and emotional regulation, thereby perpetuating academic stress [4,10].

Several studies suggest that medical students experience poorer sleep quality than many non-medical student populations, likely reflecting heavier academic workloads and stress exposure [3,9]. For example, comparative studies have reported higher PSQI scores and a greater prevalence of poor sleep quality among medical students than non-medical peers, while meta-analytic evidence indicates a consistently high burden across medical training settings [3,8]. Sleep deprivation in this group has been associated with reduced attention, impaired clinical judgment, emotional instability, and increased vulnerability to anxiety and depressive symptoms [10,11].

Clinical Workload and Night Duties

Early clinical exposure introduces additional sleep-related challenges for medical students [7]. Prolonged ward hours, early morning clinical rounds, night duties, and irregular schedules may reduce total sleep time and disrupt the circadian rhythm, the endogenous approximately 24-hour internal biological timing system that regulates sleep-wake patterns, hormone secretion, and alertness [12,13]. The normal sleep-wake cycle is synchronized by environmental cues such as light exposure and habitual schedules, promoting wakefulness during the day and sleep at night [13]. Repeated disruption of this cycle through night duties or shifting schedules may contribute to sleep restriction, daytime fatigue, and persistent circadian misalignment [12,13].

Digital Device Use and Smartphone Overuse

The widespread use of digital devices, including smartphones, tablets, laptops, and e-readers, has emerged as an important behavioral contributor to poor sleep quality among medical students. Excessive evening use of smartphones is particularly well studied and has been associated with delayed sleep onset, shorter sleep duration, and poorer subjective sleep quality [14]. Studies involving other screen-based devices have similarly shown that nighttime exposure to light-emitting screens may adversely affect sleep, circadian timing, and next-morning alertness [13]. One proposed mechanism is suppression of melatonin, a pineal hormone that normally rises in the evening to facilitate sleep initiation and regulate circadian timing. Screen light exposure, especially blue-enriched light, may disrupt circadian signaling, the transmission of time-related cues from the light-dark cycle to the body’s internal clock, thereby delaying sleepiness and shifting sleep timing [13].

In addition, cognitive and emotional engagement with digital content may increase pre-sleep arousal [14]. The reported prevalence of problematic smartphone use among students varies widely across studies and regions, and higher use has been associated with poorer PSQI scores, daytime dysfunction, anxiety, and stress [14]. Given the dual role of digital devices as educational tools and sources of distraction, targeted strategies promoting mindful and time-restricted evening use may be beneficial.

Lifestyle factors and sleep hygiene

Lifestyle behaviors and sleep hygiene practices play an important role in shaping sleep quality among medical students. Irregular sleep schedules, physical inactivity, excessive caffeine consumption, and late-night studying contribute to sleep disruption [15,16]. Sleep hygiene encompasses behaviors performed before bedtime, such as stimulant use and screen exposure, as well as environmental factors during sleep, including light, noise, and temperature [15,16].

Observational studies indicate that poor adherence to sleep hygiene recommendations is common among medical students and is associated with poor sleep quality, including longer sleep latency, shorter sleep duration, and greater daytime dysfunction [17]. In student populations, poorer sleep hygiene scores have also been linked with worse overall sleep quality outcomes [15]. Sleep hygiene is commonly assessed using multiple validated scales that evaluate behavioral, environmental, and psychological components [15,16], as summarized in Table 2.

**Table 2:** Comparison of Sleep Hygiene Scales and Commonly Assessed Components in Observational Studies (prepared by the authors based on references [15-17]). A check mark (✓) indicates that the corresponding component is included in the respective scale.

Sleep Hygiene Factors	Sleep Hygiene Index [16]	Sleep Hygiene Practices Scale [18]	Sleep Hygiene Awareness and Practices Scale [18]	Sleep Hygiene Self-Test [17]
Behavioral components				
Consumption of caffeine before bedtime	✓	✓	✓	✓
Alcohol intake	✓	✓	✓	✓
Engagement in regular physical activity	✓	✓	✓	✓
Consistency of sleep schedule	✓	✓	✓	✓
Daytime naps	✓	✓	✓	✓
Smoking / nicotine exposure	✓	✓	✓	✓
Relaxation or wind-down routine	✓	✓	✓	✓
Control of stimulus activity in bed	✓	✓		
Eating shortly before bedtime		✓	✓	✓
Restricting time spent in bed				✓
Use of sleep medication				✓
Other substances		✓	✓	✓
Environmental factors				
Light exposure	✓	✓	✓	✓
Noise level during sleep	✓	✓	✓	✓
Room temperature	✓		✓	✓
Mattress and bedding comfort	✓	✓		
Presence of a bed partner		✓	✓	
Psychological factors				
Stress/emotional state before bedtime	✓	✓	✓	✓

Psychological and psycho-emotional factors

Psychological distress, including anxiety, depression, burnout, and emotional exhaustion, is consistently associated with poor sleep quality among medical students [18]. Numerous studies demonstrate that students reporting higher levels of psychological symptoms are more likely to experience sleep disturbances [18]. The relationship between sleep and mental health is bidirectional, with sleep problems exacerbating emotional dysregulation and reduced coping capacity, while psychological distress further disrupts sleep continuity [11].

Burnout and emotional exhaustion, although less frequently quantified than anxiety and depression, follow a similar pattern and are particularly prevalent during clinical years [8,18]. These findings underscore the importance of integrated mental health support and sleep-focused interventions within medical training environments [18].

Substance use

The use of stimulants and other psychoactive substances to cope with academic demands is common among medical students. In this context, stimulants primarily refer to caffeine-containing products such as coffee, tea, caffeinated soft drinks, energy drinks, and caffeine tablets [19]. Caffeine consumption is especially common during examination periods. While moderate intake may temporarily improve alertness, excessive consumption, particularly in the late afternoon or evening, may impair sleep by blocking adenosine receptors, reducing homeostatic sleep drive, increasing sympathetic activation, delaying sleep onset, and decreasing total sleep time [19].

Alcohol use is sometimes perceived as a sleep aid because of its initial sedative effects, which may shorten sleep latency. However, alcohol subsequently disrupts normal sleep architecture, suppresses rapid eye movement (REM) sleep early in the night, increases sleep fragmentation during metabolism, and reduces restorative sleep quality [20]. Together, these substance-related behaviors may contribute to poor sleep quality and warrant targeted educational interventions [19,20].

Impact of sleep disturbances on quality of life among medical students

Sleep disturbances among medical students are associated with substantial negative effects across multiple domains of quality of life, including physical health, mental well-being, cognitive functioning, academic performance, and social relationships [3,18]. Evidence from cross-sectional studies, multicenter surveys, and meta-analyses consistently demonstrates that poor sleep quality is associated with impaired daily functioning and reduced overall well-being in this population [3]. The key findings related to these quality-of-life domains are summarized in Table 3.

**Table 3:** Impact of Sleep Disturbances on Quality of Life Domains *Abbreviations*: PSQI: Pittsburgh Sleep Quality Index; GPA: grade point average; DASS: Depression Anxiety Stress Scales; PHQ: Patient Health Questionnaire; GAD: Generalized Anxiety Disorder; WHOQOL-BREF: World Health Organization Quality of Life-BREF.

Quality of Life Domain	Key Findings in Medical Students	Representative Evidence	Study Context
Physical health	60-70% classified as poor sleepers; fatigue, daytime sleepiness, somatic complaints	PSQI-based studies [3]	Meta-analysis; multiple countries
Mental well-being	Depression (≈38–42%), anxiety (≈45–53%), high stress	PSQI correlated with DASS, PHQ, and GAD [4,18]	Saudi Arabia / Nepal; cross-sectional
Cognitive function	Impaired attention and memory; daytime dysfunction up to 86%	PSQI daytime dysfunction [10,21]	Review / China cross-sectional
Academic performance	Poor sleepers had significantly lower GPA (2.92±1.09) compared with good sleepers (3.31±1.49); p<0.0001	Maheshwari and Shaukat [22]	Pakistan; cross-sectional
Social functioning	Lower WHOQOL-BREF social domain scores	WHOQOL-BREF Social domain [23]	Multinational validation study

Physical health and daytime functioning

Several studies report that medical students classified as poor sleepers (commonly PSQI >5) experience higher levels of physical fatigue, daytime sleepiness, somatic complaints, and reduced daytime functioning compared with students reporting good sleep quality [3]. Across PSQI-based studies, approximately 60%-70% of participants in several cohorts were categorized as poor sleepers, with higher frequencies of headaches, gastrointestinal discomfort, low energy, and impaired daily performance [3,4,5].

Poor sleep quality has also been associated with increased reliance on stimulants, reduced physical activity, and lower participation in routine academic and social activities [6]. Collectively, these symptoms may impair energy levels, functional capacity, and self-perceived health status, thereby contributing to reduced physical quality of life [3,6].

Mental health and psychological well-being

Poor sleep quality demonstrates one of the most consistently reported associations with adverse mental health outcomes among medical students [7,18]. Multiple studies across diverse regions report higher prevalence of depression (approximately 38% to 42%), anxiety (45% to 53%), and high perceived stress among students experiencing sleep disturbances or poor sleep quality [4,18]. In a large cross-sectional study, PSQI scores showed significant positive correlations with validated measures of depression, anxiety, and stress, indicating that worsening sleep quality parallels increasing psychological distress [4].

The relationship between sleep disturbance and mental health is bidirectional, as sleep problems may worsen emotional dysregulation and impair coping capacity, while psychological distress may further disrupt sleep continuity [11]. Persistent sleep disturbances may therefore contribute to burnout, emotional exhaustion, and reduced psychological resilience during medical training [8,11].

Cognitive function and daytime performance

Sleep is essential for memory consolidation, attention regulation, and executive functioning, all of which are critical for medical education. Experimental sleep research has demonstrated that sleep deprivation leads to reduced attention span, impaired working memory, slower cognitive processing, and decreased decision-making accuracy [10].

Among medical students, subjective cognitive impairment is frequently reported in association with poor sleep quality, particularly in the domains of concentration, alertness, and information retention [10,21]. The daytime dysfunction component of the PSQI, reflecting difficulties in maintaining alertness during daily activities, has been reported in as many as 86% of poor sleepers in some cohorts [21]. These impairments may adversely affect learning efficiency, clinical reasoning, and patient-care performance [10].

Academic performance

Multiple studies directly quantify the relationship between sleep quality and academic outcomes in medical students. Poor sleep quality has been consistently associated with lower grade point averages [22], reduced examination performance, increased absenteeism, and diminished academic engagement [10].

Maheshwari and Shaukat demonstrated that medical students classified as poor sleepers had a significantly lower mean grade point average (2.92±1.09) compared with good sleepers (3.31±1.49), with strong statistical significance (p<0.0001) [22]. Similarly, Alsaggaf and colleagues reported that 73.7% of participating Saudi medical students were classified as poor sleepers, and this was significantly associated with negative academic outcomes [7].

Although some studies suggest that the relationship between sleep quality and academic performance may be partially mediated by psychological stress and depressive symptoms [7,24]. The overall evidence supports an association between poor sleep quality and impaired academic achievement [7].

Social functioning and interpersonal relationships

Sleep disturbances are also associated with poorer social functioning and interpersonal relationships among medical students. Medical students with poor sleep quality consistently report lower scores in social domains of quality-of-life assessments, including reduced satisfaction with personal relationships and decreased social participation [23].

Sleep deprivation has been linked to irritability, emotional lability, social withdrawal, and reduced empathy, which may impair peer interactions and teamwork [11]. Lower social functioning may further exacerbate psychological distress and contribute to a reinforcing cycle of poor sleep and reduced quality of life [11].

Sleep disturbances, metabolic health, and cardiovascular risk

Chronic sleep disturbances have been increasingly linked to adverse metabolic and cardiovascular outcomes, primarily in the general population and cohort studies. Insufficient sleep duration and poor sleep quality are associated with hormonal dysregulation [1], including alterations in cortisol secretion, impaired glucose tolerance, reduced insulin sensitivity, increased sympathetic nervous system activity, and systemic inflammation. These physiological changes may contribute to the development of obesity, metabolic syndrome, type 2 diabetes mellitus, and cardiovascular disease [1,6,25,26].

Epidemiological evidence from predominantly non-student populations consistently demonstrates that short sleep duration is associated with increased risk of overweight, obesity, and progressive weight gain over time [25,26]. Sleep deprivation has also been shown to impair appetite regulation through the dysregulation of leptin and ghrelin [25], promoting increased caloric intake and reduced energy expenditure. Although much of this evidence originates from general-population studies, medical students may represent a uniquely vulnerable subgroup because of chronic sleep restriction during formative academic and professional years.

Emerging data indicate that persistent sleep disturbances may contribute to long-term cardiometabolic risk, including hypertension and endothelial dysfunction [6]. Given the high prevalence of poor sleep quality among medical students, early exposure to these risk factors may have implications for future cardiovascular health, suggesting the potential value of preventive strategies within medical education.

Screening, counseling, and curriculum-level interventions

Given the high prevalence and significant consequences of sleep disturbances among medical students, early identification and targeted intervention may be considered priorities within medical training institutions. Routine screening using validated tools such as PSQI [2] and the Insomnia Severity Index (ISI) [27] allows for early detection of sleep problems, even among students who may not voluntarily report symptoms.

Integrating sleep health education into medical curricula represents a promising strategy to improve awareness and promote healthier sleep behaviors. Studies evaluating sleep education interventions among medical students demonstrate improvements in sleep knowledge [10], attitudes, and self-reported sleep hygiene practices, although effects on sleep quality may vary [2]. Counseling services addressing stress management, anxiety, depression, and burnout may also be valuable, given the strong bidirectional relationship between psychological distress and sleep disturbance [11,18].

At the institutional level, policy-driven interventions aimed at optimizing academic schedules, limiting excessive workload, and discouraging the normalization of sleep deprivation within medical culture may contribute to sustainable improvements in student well-being [3,5]. Faculty awareness and supportive learning environments may play an important role in fostering a culture that recognizes sleep as a fundamental component of professional competence and long-term physician health.

Limitations

This narrative review has several limitations. As a non-systematic synthesis, the review did not employ a formal risk-of-bias assessment or quantitative meta-analysis. Variability in study design, geographic representation, and PSQI cutoff thresholds across studies may influence prevalence estimates. The possibility of publication bias and language bias cannot be excluded, as studies with significant findings and English-language publications may be more likely to be indexed and retrieved. Many included studies also relied on self-reported instruments, particularly PSQI, which may be subject to recall bias and limited objective sleep assessment. Additionally, most included studies were cross-sectional, limiting causal inference between sleep disturbances and quality-of-life outcomes.

Despite these limitations, this review has several strengths, including the synthesis of recent international literature, focus on a high-risk and under-addressed population, and integration of multiple domains of impact, including physical, psychological, cognitive, academic, and social well-being. The findings highlight practical implications for medical schools, suggesting that early screening, sleep-health education, counseling support, and workload-sensitive institutional strategies may help improve student well-being. Future longitudinal and interventional research is needed to clarify temporal relationships and evaluate the effectiveness of such strategies.

## Conclusions

Sleep disturbances are highly prevalent among medical students and appear to arise from a complex interplay of academic pressure, demanding clinical responsibilities, maladaptive lifestyle behaviors, psychological distress, and inadequate sleep hygiene. Evidence synthesized in this narrative review suggests that poor sleep quality is consistently associated with fatigue, impaired daytime functioning, depression, anxiety, burnout, and reduced academic performance. In addition to cognitive and psychological consequences, broader literature indicates that persistent sleep disturbances may contribute to long-term metabolic and cardiovascular risks, although direct longitudinal evidence in medical students remains limited.

Given these findings, early identification and management of sleep problems may be beneficial within medical training environments. Routine screening using validated instruments, accessible counseling services, structured sleep-health education, and institutional efforts to reduce excessive workload represent potential strategies that could help mitigate adverse outcomes. Promoting healthy sleep practices during medical training may improve quality of life, academic performance, and long-term well-being while helping to foster a healthier and more resilient future healthcare workforce.
